# Novel Allosteric Effectors Targeting Human Transcription Factor TEAD

**DOI:** 10.3390/ijms24109009

**Published:** 2023-05-19

**Authors:** Mayar Tarek Ibrahim, Gennady M. Verkhivker, Jyoti Misra, Peng Tao

**Affiliations:** 1 Department of Chemistry, Center for Research Computing, Center for Drug Discovery, Design, and Delivery (CD4), Southern Methodist University, Dallas, TX 75205, USA; mayarm@smu.edu (M.T.I.); ptao@smu.edu (P.T.); 2 Graduate Program in Computational and Data Sciences, Schmid College of Science and Technology, Chapman University, Orange, CA 92866, USA; 3 Department of Biomedical and Pharmaceutical Sciences, Chapman University School of Pharmacy, Irvine, CA 92618, USA; 4 Department of Biological Sciences, University of Texas at Dallas, Richardson, TX 75080, USA; jyoti.misra@utdallas.edu

**Keywords:** Hippo pathway, TEAD4/YAP1 interaction, chemical modification, Markov state model, free energy perturbation

## Abstract

The Hippo pathway is an evolutionary conserved signaling network involved in several cellular regulatory processes. Dephosphorylation and overexpression of Yes-associated proteins (YAPs) in the Hippo-off state are common in several types of solid tumors. YAP overexpression results in its nuclear translocation and interaction with transcriptional enhanced associate domain 1-4 (TEAD1-4) transcription factors. Covalent and non-covalent inhibitors have been developed to target several interaction sites between TEAD and YAP. The most targeted and effective site for these developed inhibitors is the palmitate-binding pocket in the TEAD1-4 proteins. Screening of a DNA-encoded library against the TEAD central pocket was performed experimentally to identify six new allosteric inhibitors. Inspired by the structure of the TED-347 inhibitor, chemical modification was performed on the original inhibitors by replacing secondary methyl amide with a chloromethyl ketone moiety. Various computational tools, including molecular dynamics, free energy perturbation, and Markov state model analysis, were employed to study the effect of ligand binding on the protein conformational space. Four of the six modified ligands were associated with enhanced allosteric communication between the TEAD4 and YAP1 domains indicated by the relative free energy perturbation to original molecules. Phe229, Thr332, Ile374, and Ile395 residues were revealed to be essential for the effective binding of the inhibitors.

## 1. Introduction

**Figure 1:**
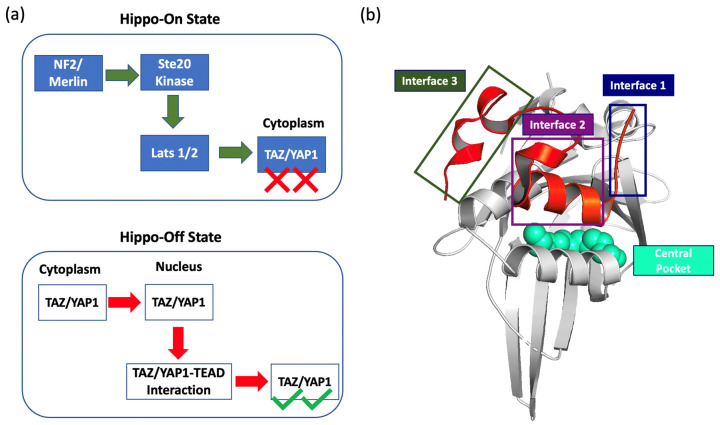
The Hippo pathway is regulated through phosphorylation of TAZ/YAP1 coactivators. (**a**) The Hippo-on state is initiated by the sequential phosphorylation process of NF2, Ste20, and Lats 1/2, and leads to the phosphorylation and the degradation of TAZ/YAP1 coactivators in the cytoplasm. The Hippo-off state involves the dephosphorylation of TAZ/YAP1 coactivators followed by their translocation to the nucleus where they interact with TEAD, resulting in their overexpression. (**b**) Three interfaces of TAZ/YAP1-TEAD as key interaction sites and the TEAD4 central pocket represented as spheres (PDB ID: 5OAQ).

The Hippo signaling network is an evolutionary conserved pathway that is critical for several essential physiological processes such as organ size control, cellular proliferation and differentiation, tissue growth, immune homeostasis, and apoptosis [1,2,3,4]. Activation of the Hippo pathway leads to phosphorylation of the paralogous transcriptional coactivators: YAP1 (yes-associated protein 1) and TAZ (transcriptional coactivator PDZ-binding motif). The YAP1/TAZ complex can be phosphorylated by the large tumor suppressor (Lats 1/2) kinase (Figure 1a), which in turn is phosphorylated and activated by the mammalian Ste20-like kinase (MST1/2) with facilitation from SAV1 and MOB1A/B and the NF2/Merlin-Kibra tumor suppressor complex [5,6].

When the Hippo pathway is active, the phosphorylated YAP1/TAZ complex is sequestered in the cytoplasm, where it is ubiquitinated and degraded [3]. On the other hand, when Hippo signaling is inactive, the YAP1/TAZ complex becomes unphosphorylated and can be translocated into the nucleus, where it interacts with transcriptional enhanced associate domain (TEAD) transcription factors (Figure 1a).

The TEAD transcription factors contain an N-terminal DNA-binding TEA domain and a C-terminal YAP binding domain (YBD) [7,8]. The YBD found in the four isoforms of TEAD, TEAD1-4, are structurally homologous with 75% identical residues and 25% similar residues [9]. The interaction between the YAP1/TAZ complex and TEAD affects several downstream genes involved in the cellular proliferation and survival [10]. The overexpression of YAP1/TAZ coactivators is common in several solid tumors by not only initiating the tumor growth but also maintaining the continued growth of the tumor [3]. This is attributed to the role that YAP1 activation plays in affecting the tumor microenvironment, inducing resistance to chemotherapy, and performing as an intrinsic oncogenic driver [11,12,13]. Therefore, targeting TAZ1/YAP1-TEAD interaction offers a promising cancer therapeutic approach.

The discovered inhibitors targeting TAZ1/YAP1-TEAD interaction can be classified into direct inhibitors and allosteric inhibitors [3]. The direct inhibitors, known as protein–protein inhibitors (PPIs) [14,15,16,17] disrupt the interaction between TEAD and YAP1 by binding to one of the three interaction interface sites. On the contrary, the allosteric inhibitors bind to the central palmitate-binding pocket of TEAD (Figure 1b) [3]. The allosteric inhibitors can be further divided into covalent binders [6,18,19,20,21], interacting with the conserved cysteine residue in the hydrophobic central pocket, or non-covalent binders [22,23,24]. The number of discovered allosteric inhibitors exceeds the number of direct inhibitors because of the extensive large shallow interaction interface between TEAD and YAP1 with an area larger than 1000 Å^2^ [25]. Hence, targeting the central pocket provides a tangible approach to inhibiting YAP1 transcriptional activity. Accordingly, screening of a DNA-encoded library against the TEAD central pocket was performed as follows. A DNA-encoded chemical library comprises a vast collection of molecules, wherein each molecule is associated with DNA tags that serve as barcodes [26]. These libraries enable the screening of significantly larger compound collections, resulting in a higher success rate for the identification of potential hits. We used the DEL-Open 4.0 library from Wuxi AppTec with GST-tagged wild-type TEAD4 and TEAD4 whose C360A mutant cannot be palmitoylated. After screening, the compounds that were specifically enriched in the TEAD4 C360A but not in the wild-type TEAD were identified. The screening resulted in 50 compounds. All 50 compounds belonged to a same sub-library, indicating a common structure. The top six compounds among these 50 compounds formed an analog series and represented non-covalent allosteric inhibitors of TEAD4 (credit to Jyoti Misra, [Unpublished results]).

**Figure 2:**
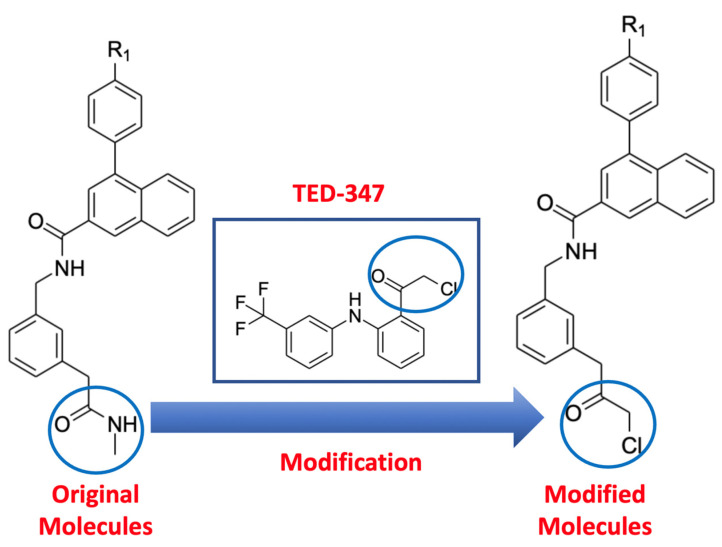
Structural modification of the experimentally obtained ligands targeting the central pocket of TEAD4, the modified moieties in the original molecules, TED-347, and the modified moieties are circled in blue. The secondary methyl amide was replaced by a chloromethyl ketone group, inspired by the chemical structure of the TED-347 ligand [6].

In this work, we studied the effect of the binding of the experimentally identified inhibitors using various computational tools including molecular dynamics simulations, molecular mechanics with generalized Born and surface area solvation (MM/GBSA), and a Markov state model (MSM). Moreover, inspired by the chemical structure of the TED-347 inhibitor [6], modification of the experimentally discovered small molecules was performed by replacing the secondary methyl amide with chloromethyl ketone (Figure 2). This modification was expected to change the protein–protein interaction between TEAD4 and YAP1 protein, similar to the effect from the TED-347 compound. The impact on the conformational space of the protein from the original and the modified molecules’ binding to the TEAD4 central pocket was inferred by using the dimensionality reduction technique time-independent component analysis (t-ICA) and the Markov state model (MSM). In addition, the difference in the allosteric communication between the two proteins was evaluated by the difference in binding free energy, ∆∆G, to measure the effect of the ligands’ binding on the strength of the protein–protein interaction between TEAD4 and YAP1. The modified molecules affected the binding affinity between TEAD4 and YAP1 proteins to an extent exceeding the original molecules obtained from the experimental screening.

## 2. Results

### 2.1. Molecular Docking of Original and Modified Molecules

Molecular docking was conducted using the FRED package [26,27,28] to compare the binding affinities of the six experimentally identified ligands and their corresponding modified counterparts. The docking scores were assessed using Chemgauss4, and the top-ranked poses were analyzed (Table 1). Among the modified ligands, five out of six exhibited higher docking scores compared to their original counterparts. However, Ligand 1 from the modified molecules showed a slightly lower docking score of −0.8 kcal/mol compared to the original molecule. The difference in Chemgauss4 scores between the original and modified ligands was generally minimal, ranging from −0.02 to −0.97 kcal/mol. This suggests that the introduced modification, which involved replacing the secondary methyl amide with chloromethyl ketone, had no significant impact on the binding of the ligands to the TEAD4 central pocket.

### 2.2. The Stability and the Binding Affinity of the Original and Modified Ligands

The experimentally identified molecules from the screening of the DNA-encoding library against the central pocket of TEAD4 were computationally modified by replacing the secondary methyl amide with chloromethyl ketone. This modification was inspired by the structure of the TED-347 inhibitor [6]. The stability of the generated binding modes was investigated by performing 150 ns molecular dynamics simulations of the top poses of the original and modified molecules, identified from molecular docking using the FRED package [27,28,29].

**Figure 3:**
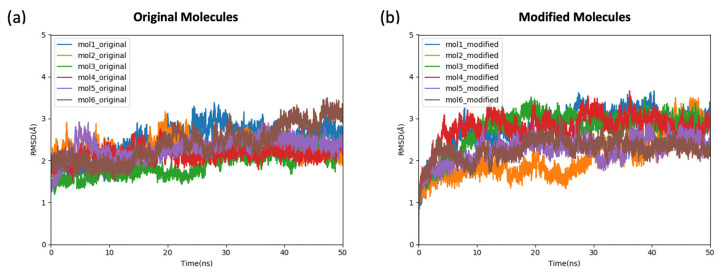
The root-mean-square deviation (RMSD) values of simulation of 12 protein–ligand complexes subjected to simulations. For clarity, the RMSD of one of the three 50 ns replicas is plotted for each complex. (**a**) RMSD values of the simulation of complexes including original experimentally obtained ligands; (**b**) RMSD values of the simulation of complexes including modified ligands.

Root mean square deviation (RMSD) values were calculated to assess the convergence of the trajectories using the first frame as the reference structure. The RMSD values from all three 50 ns trajectories fluctuated between 1 and 3 Å for each of the experimentally identified and computationally modified ligands, indicating the stability of the protein–ligand binding in both cases (Figure 3).

**Figure 4:**
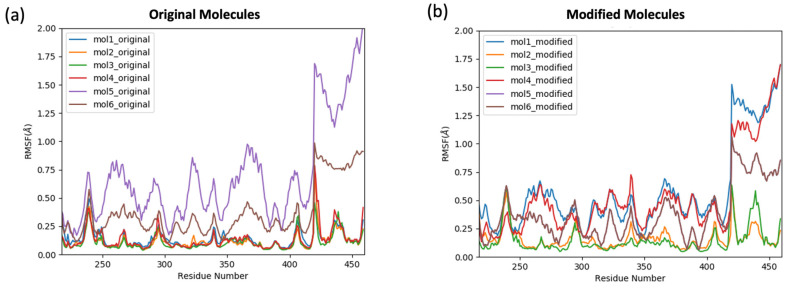
The root-mean-square fluctuation (RMSF) of the trajectories of the protein bound with ligand with regard to the crystal structure of the protein. (**a**) RMSF of the protein trajectories bound with original molecules; (**b**) RMSF of the protein trajectories bound with the modified molecules.

To explore the effect of the ligands’ binding on the residual fluctuation of the TEAD4-YAP1 complex, the root mean square fluctuation (RMSF) of the backbone atoms was calculated with reference to the crystal structural with no bound ligand (Figure 4). High RMSF fluctuations were observed in the YAP1 protein, 210 to 250 residues, in both original (Figure 4a) and modified ligands (Figure 4b), with larger deviations in the modified ligands. This indicated that the binding of the ligands to the TEAD4 central pocket induced high flexibility in the YAP1 structure. On the other hand, the fluctuations of TEAD4 residues during the simulation ranged between 0 and 0.5 Å for the modified ligand, whereas for the original molecules, the fluctuation ranges extended slightly more as between 0 and 0.75 Å. These small ranges in fluctuation in the TEAD4 protein suggested the lower sensitivity of the TEAD4 structure upon the binding of the ligands, compared to the YAP1 protein.

The MM/GBSA method was used to calculate the binding free energy of the 12 ligands against the TEAD4 central hydrophobic pocket. The reported binding free energies of the six original and the six modified ligands were the average of the binding energies calculated based on each of the three independent 50 ns trajectories. The binding free energies of the original ligands ranged between −56.33 and −65.34 kcal/mol, and the binding free energy of the modified ligands ranged between −49.87 and −62.73 kcal/mol (Table 2). Most of the original ligands, except for ligand 4, were found to have more favorable binding free energies to the TEAD4 central palmitate-binding pocket than the modified ligands. The difference in binding energies between the original and the corresponding modified ligands ranged between −0.31 and −6.46 kcal/mol. This suggested that the modified ligands could bind at the same pocket with similar binding affinity to the original ligands.

### 2.3. The Effect of the TEAD4 Ligands on the Conformational Space of the Protein

#### 2.3.1. Time-Independent Component Analysis (t-ICA)

**Figure 5:**
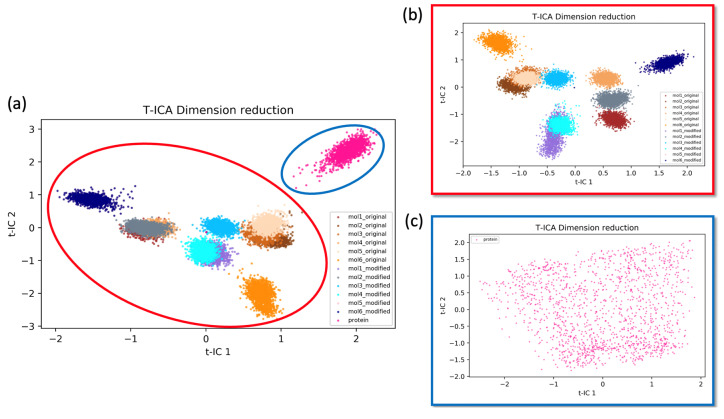
Time-independent component analysis (t-ICA) using Cα distances as features to study the effect of ligands’ binding on the conformational space of the TEAD4-YAP1 complex. (**a**) The distribution of the simulations of 12 complexes bound with the ligands and the complex without ligand; (**b**) the distribution of the simulations of the 12 complexes bound with the ligands; (**c**) the distribution of the simulations of the complex without ligand.

The effect of the ligands’ interaction with TEAD4 on the conformational space of TEAD4 was investigated using the dimensionality reduction method t-ICA. Geometric features in the form of pairwise Cα distances were employed to analyze the protein structure independently, excluding the bound ligand for t-ICA. The latent space representing the conformational space of the protein was built using the two most dominant vectors of t-ICA, t-IC1 and t-IC2, for the complex structures of TEAD4-YAP1 bound with the ligands, as well as the complex structure without ligand (Figure 5a). The complex structure with ligand explored an entirely different conformational space compared to the complexes without ligand, indicating that the binding of ligands resulted in a significant impact on the distribution of protein in the conformational space. To better compare the effect of ligand binding on the conformational change, t-ICA was performed based on the complex structures with ligands (Figure 5b) and based on the complex structures without ligand (Figure 5c).

Overall, the distributions of the TEAD4-YAP1 complexes bound with different ligands showed significantly different patterns. Specifically, the complexes bound with the original ligands covered a different area from those bound with the modified ligands, suggesting that the modified ligands exerted a distinct impact on the protein conformational distribution from the original ligands. The distributions of mol3_original and mol5_original in the reduced dimensional space covered the same area. Similarly, mol1_modified and mol4_modified explored a similar conformational space. The distribution of the TEAD4-YAP1 structure without ligand evenly covered a wide range of reduced dimensional space (Figure 5c). This suggested that the TEAD4-YAP1 complex is flexible and explores the accessible conformational space with evenly distributed probabilities. The binding with different ligands could exert a significant impact that limits the conformational space explored by protein.

#### 2.3.2. Markov State Model Analysis

**Figure 6:**
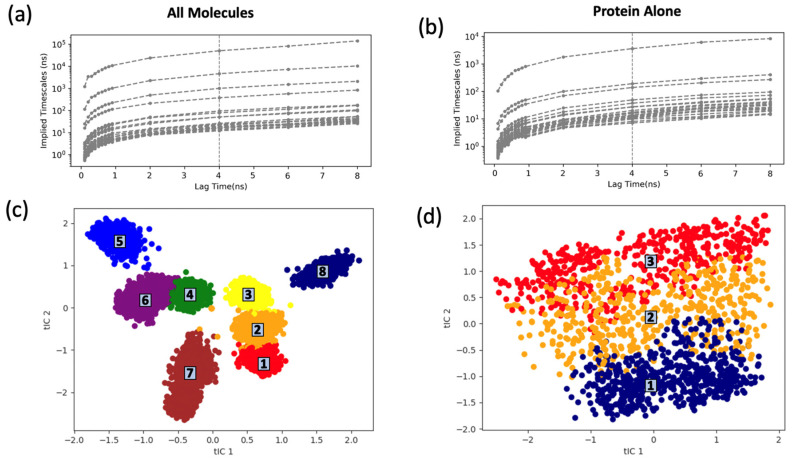
Implied time scale and the constructed macrostates’ conformational space for Markov State Model (MSM) building based on t-ICA analysis. (**a**) Implied time scale analysis based on the simulations of TEAD4-YAP1 complexes bound with ligands; (**b**) implied time scale analysis based on the simulation of the protein complex without ligand. We chose 4 ns as the lag time for the MSM for both cases; (**c**) the conformational space of the eight macrostates generated from the trajectories of the holo structure of TEAD4/YAP1 complex. The 8 macrostates are numbered and colored differently for easier analysis; (**d**) the conformational space of the three macrostates generated from the trajectories of the apo structure. The 3 macrostates are numbered and colored differently.

The distribution of the TEAD4-YAP1 complex without ligand and the complexes bound with ligands in the reduced dimensional space based on t-ICA, generated from the three replicas of each configuration with a total of 150 ns molecular dynamics simulations, were clustered into 90 microstates using the k-means clustering method. The transition probabilities among these microstates were calculated to choose an appropriate number of macrostates and the proper lag time. Based on the convergence of the implied time scale, 4 ns was chosen as the lag time to build the MSM in both cases (Figure 6). Eight macrostates were selected based on the implied time scale constructed for the protein complexes bound with ligands (Figure 6a). In our analysis, we performed a 10-fold cross-validation on the data divided into an 80% training set and a 20% test set. The results indicated that the MSM with eight macrostates yielded the highest GMRQ scores (Figure S1a). For the protein complex without ligand, we chose to use a total of three macrostates for the MSM (Figure 6b), which was found to be the optimal number based on the same analysis, showing the highest GMRQ scores for the MSM with three macrostates (Figures S1b and S2). 

The distribution of the eight macrostates identified in the MSM analysis revealed that the binding of the original and the modified ligands could significantly affect the distribution of the protein in conformational space (Figure 6c). These macrostates were distinct from each other through either gap or clear boundary. On the contrary, the distributions of the three macrostates in the MSM for the protein complex without ligand overlapped with each other without clear boundary (Figure 6d).

**Figure 7:**
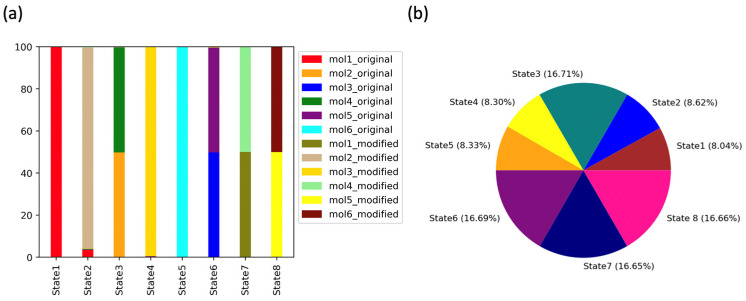
Adjusted population distribution of the eight MSM macrostates of the complexes with ligands. (**a**) Stacked bar chart distribution; (**b**) pie chart distribution.

The distribution of the adjusted populations [30] constructed from the eight macrostates obtained from the MSM analysis for the protein complexes bound with ligands was calculated and plotted (Figure 7). Macrostates 1, 2, 4, and 5 were mainly formed of a single configuration of a complex structure bound with a specific ligand. Macrostate 1 was mainly composed of mol1_original (complex bound with Original 1 ligand listed in Table 1), macrostate 2 was mainly composed of mol2_modified (complex bound with Modified 2 ligand listed in Table 1), macrostate 4 was mainly composed of mol3_modified, and macrostate 5 was mainly composed of mol6_original (Figure 7a).

In contrast, macrostates 3, 6, 7, and 8 were composed of two different configurations. Macrostate 3 was composed of mol2_original and mol4_original, macrostate 6 was composed of mol3_original and mol5_original, macrostate 7 was composed of mol1_modified and mol4_modified, and macrostate 8 was composed of mol5_modified and mol6_modified. The clear separation of the complexes bound with different ligands among the macrostates supported the hypothesis that the simulations of these complexes sampled different conformational spaces of the protein complex. Accordingly, binding with Original 2 and 4 ligands led to the similar conformational space sampled by the TEAD4-YAP1 complex because mol2_original and mol4_original belong to macrostate 3. Similarly, Original 3 and 5 ligands displayed a similar impact on the TEAD4-YAP1 complex conformational space. The Modified 1 and 4 ligand pair and Modified 5 and 6 ligand pair were the two pairs of ligands with a similar impact on protein complex.

In addition to the investigation of the conformational space occupied by the macrostates, MSM analysis showed which macrostates were more abundant, indicating a better stability of the associated TEAD4-YAP1 complex (Figure 7b). The structures occupying state 3, state 6, state 7, and state 8 were more abundant and therefore likely to be more stable.

The adjusted population’s distribution of the 3 macrostates generated from the MSM analysis of the TEAD4-YAP1 protein complex with no bound ligand showed a nearly equal distribution of the three macrostates, with 39.04% for macrostate 1, 30.84% for macrostate 2, and 30.12% for macrostate 3.

**Figure 8:**
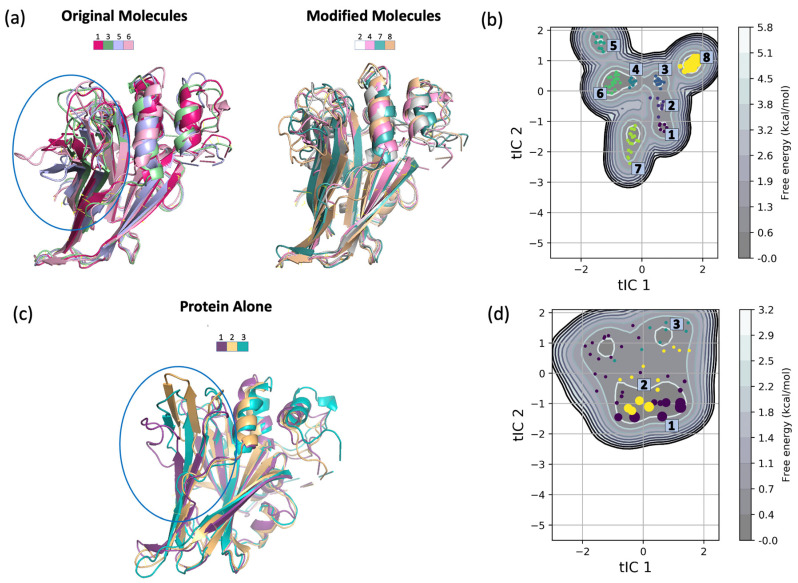
Representative structures and free energy landscape of the generated macrostates. Each macrostate is colored differently for easier comparison. The part with more structural changes upon the binding of the ligands is circled in blue. (**a**) Representative structures of the macrostates associated with the original molecules (States 1, 3, 5, and 6) and the macrostates associated with the modified molecules (States 2, 4, 7, and 8); (**b**) free energy landscape of the eight macrostates constructed from the trajectories of the protein with bound ligand; (**c**) representative structures of the three macrostates generated from the protein alone; (**d**) free energy landscape of the three macrostates.

Representative structures for the constructed macrostates from the MSM analysis were extracted from the production trajectories and aligned to perform in-depth structural analysis and to deduce differences from the free energy landscape (Figure 8). The original molecules and the modified molecules were found to induce significant structural changes to the β-sheets and the loops adjacent to the palmitate-binding pocket (Figure 8a). On the other hand, the same secondary structures in the protein with no ligand bound were subjected to fewer changes (Figure 8c). In addition, the structural integrity of the YAP1 protein was affected by the binding of the ligands, where at least one of the two small α-helices found near the C-terminal of the protein unraveled. This explained the high fluctuations of the YAP1 residues observed in the RMSF curve (Figure 4). This unwinding of the α-helices was not observed in the protein with no bound ligand.

The free energy landscape of the generated macrostates supported the previous analysis that the binding of the ligands resulted in higher structural changes, thus higher energies (Figure 8b). The distribution of the macrostates showed that macrostates 1, 2, 3, 4, and 6 shared the same energy basin. This indicated the close conformation of these five macrostates as indicated by their close energetics.

States 5, 7, and 8 were on the outskirts of the energy landscape and occupied their unique energy basin. This suggested that the binding of mol6_original (most abundant configuration in state 5), mol1_modified and mol4_modified (most abundant configuration in state 7), and mol5_modified and mol6_modified (most abundant configuration in state 8) induced more significant conformational change as indicated by the higher energetics.

On the other hand, the free energy landscape of the protein alone was associated with lower energy values and more disperse distribution (Figure 8d).

### 2.4. Impact of Ligands’ Binding on the Relative Free Energy Perturbation

**Figure 9:**
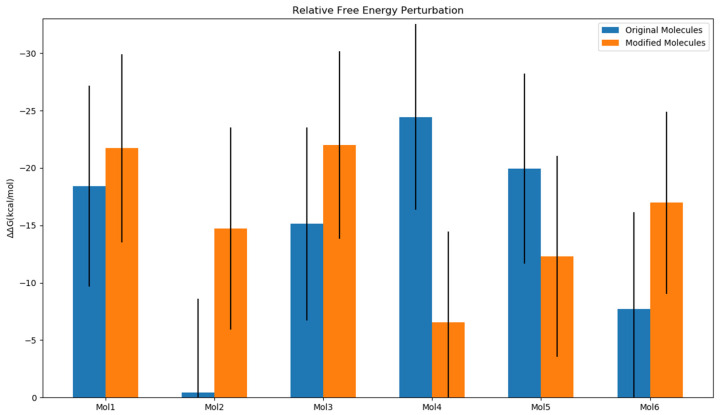
The change in the TEAD4-YAP1 binding free energies (ΔΔG) upon binding with ligands.

The binding free energy between TEAD4 and YAP1 domains was calculated using the MM/GBSA method to investigate the effect of the ligand binding on the interaction strength between the TEAD4 and YAP1 domains. Figure 9 illustrates the variation in the calculated binding free energies of the TEAD4-YAP1 complex upon binding with ligands, relative to the calculated binding free energy in the absence of ligands (represented as ∆∆G). For all ligands in this study, the presence of a ligand led to more favorable binding affinity between TEAD4 and YAP1. Because the ligands bound with TEAD4, this suggested that the binding with TEAD4 could stabilize the TEAD4 and YAP1 complex.

Four modified ligands, mol1_modified, mol2_modified, mol3_modified, and mol6_modified, led to increased TEAD4-YAP1 binding affinity more favorably than their corresponding original ligands. These more favorable changes showed that the proposed chemical modification of the original ligands (Figure 2) significantly enhanced the stability of the TEAD4-YAP1 complex. The variations in ∆∆G values between the original and modified molecules ranged from −3.29 kcal/mol for mol1 to −14.31 kcal/mol for mol2. Notably, the binding of mol2_original had minimal impact on the binding strength between TEAD4 and YAP1, indicating that the difference between the apo and holo structures of the protein was negligible (Table S1 and Figure 9). This implied that most modified ligands affected the TEAD4 and YAP1 interaction more significantly than the original ligands. The chemical modification led to enhanced cooperative effects among TEAD4, YAP1, and the ligands in these cases. For ligands 4 and 5, the chemical modification of the original ligands led to a significantly deceasing stabilization effect on the TEAD4-YAP1 complex. Specifically, Original Molecules 4 and 5 resulted in positive ΔΔG values as 17.90 and 7.63 kcal/mol, respectively (Figure 9).

**Figure 10:**
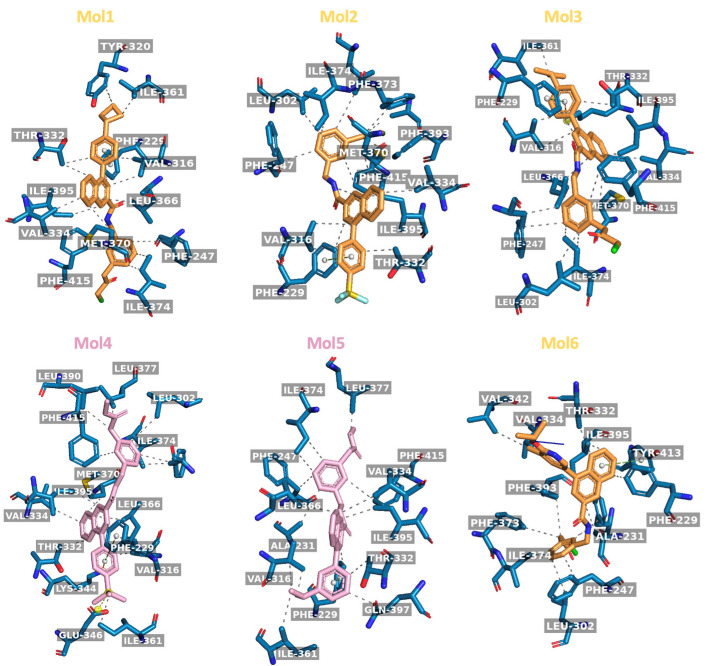
The binding modes of the ligands associated with more favorable binding affinity between TEAD4 and YAP1. The original ligands are colored in pink, and the modified ligands are colored in yellow. Figures are generated using the PLIP package [31].

We further investigated the binding modes for TEAD4 with the ligands associated with more enhanced favorable binding affinities with TEAD4, including Modified 1, 2, 3, and 6 ligands, Original 4 and 5 ligands (Figure 10). All six ligands had an extensive interaction network with the central palmitate-binding pocket of TEAD4. All six ligands formed hydrogen bonds with Phe229, Thr332, Ile374, and Ile395 in TEAD4. Several other residues also showed strong interactions with five of these ligands: Val316 (except for Modified 6), Phe247 (except for Original 4), Phe415 (except for Modified 6), and Val334 (except for Original 5) (Figure 10). Met370 was a common residue in the interaction of the four ligands associated with more enhanced binding affinity between TEAD4 and YAP1 domains, except for ligands 5 and 6.

It was noticeable that the RMSF of mol5_original was distinct from the other ligands (Figure 4). The binding mode analysis showed that mol5_original and mol5_modified formed a unique hydrogen bond with Gln397, which was absent in the other molecules. The Baker–Hubbard analysis of the intramolecular hydrogen bond network and intermolecular hydrogen bond network between TEAD4 and YAP1 revealed that Gln397 formed a hydrogen bond with Tyr320 (Figure S3a). On the contrary, Gln397 formed a hydrogen bond with Ile411 in the other complexes (Figure S3b). This key difference in the ligands’ binding to the TEAD4 central pocket and the associated changes in the hydrogen bond patterns could contribute to the large RMSF fluctuation of mol5 observed in the simulations.

By comparing them with the binding modes of these six ligands, the remaining six ligands associated with less enhanced binding affinity showed similar binding modes but lacked the main interactions with Phe229, Thr332, Ile374, and Ile395 in TEAD4 (Figure S4). The six ligands with less enhanced binding affinity did not form hydrogen bond interactions with the above four residues simultaneously. This implied the importance of forming these four hydrogen bonds for maximum stabilization of the TEAD4-YAP1 complex.

### 2.5. Formatting of Mathematical Components

The root-mean-square deviation (RMSD) of the protein structure was calculated as

(1) $$RMSD=\sqrt{\frac{1}{N}\sum_{i=1}^{N}({Ur}_{i}-r_{i}^{ref}{)}^{2}}$$

where N is the total number of atoms, $r_{i}$ is the coordinate of atom I, $r_{i}^{ref}$ is the coordinate of atom i in the reference structure, and U is the best-fit rotational matrix to align a given structure onto the reference structure.

The root mean square fluctuation (RMSF) of the protein structure in the simulation was calculated as

(2) $$RMSF=\sqrt{\frac{1}{T}\sum_{t=1}^{T}(r_{t}-\overset{-}{r}{)}^{2}}$$

where T is the number of frames.

Binding free energies using the MM/GBSA method were calculated as

(3) $$\begin{array}{c} {△G}_{bind}={△G}_{bind,vacuum}+{△G}_{solv,complex}-{(△G}_{solv,ligand}\\ +{△G}_{solv,receptor}) \end{array}$$

where ${△G}_{bind,vacuum}$ is the binding free energy in a vacuum, ${△G}_{solv,complex}$ is the solvation energy of the complex, ${△G}_{solv,ligand}$ is the solvation energy of the ligand, and ${△G}_{solv,receptor}$ is the solvation energy of the receptor.

Transition probabilities *T_ij_* between macrostates *i* and *j* were calculated as

(4) $$T_{ij}\left(\tau \right)=\frac{C_{ij}}{\sum_{k}C_{ik}}$$

where $C_{ij}$ is the count of the trajectories’ transition from a state i to a state j within a certain lag time τ.

Eigenvalue decomposition of the transition probability matrix *T* is represented as

(5) $$T\left(\tau \right)\phi_{i}=\lambda_{i}\phi_{i}$$

where $\phi_{i}$ represents the eigenvector and $\lambda_{i}$ represents the corresponding eigenvalue.

The standard deviation, σ, of binding free energies using the MM/GBSA method listed in Table 2 were calculated as

(6) $$\sigma =\sqrt{\frac{\sum_{i=1}^{N}{\left|x_{i}-\bar{x}\right|}^{2}}{N}}$$

where N is the total number of samples, which was three for the three independent trajectories for each system, *x_i_* is the binding free energy calculated using each trajectory, and $\bar{x}$ is the averaged binding free energy for each system.

The error bars in Figure 9 for the change in the TEAD4-YAP1 binding free energies (ΔΔG) upon binding with ligands, $\sigma_{\Delta \Delta G}$, were calculated as the square root of the summation of the variances of the TEAD4-YAP1 binding free energies with and without ligand binding as ${\left(\sigma_{\Delta G\_bound}\right)}^{2}$ and ${\left(\sigma_{\Delta G\_free}\right)}^{2}$, respectively:

(7) $$\sigma_{\Delta \Delta G}=\sqrt{{\left(\sigma_{\Delta G\_bound}\right)}^{2}+{\left(\sigma_{\Delta G\_free}\right)}^{2}}$$

## 3. Discussion

The protein–protein interaction between TEAD4 and YAP1 in the Hippo-off state harnessed the overexpression of the YAP1 and TAZ coactivators, which was found to be common in several types of solid tumors [3]. The allosteric effectors binding to the TEAD4 central pocket were the most potent inhibitors for the TEAD4-YAP1 interaction [18,19,20,21,22,23,24].

Screening of a DNA-encoding library against the TEAD4 hydrophobic pocket resulted in identifying six ligands with a common secondary methyl amide substituent. Inspired by the structure of TED-347 [6], chemical modification was introduced by replacing the secondary methyl amide with chloromethyl ketone in these six original ligands. Molecular docking of the six original and the six modified ligands was performed followed by 150 ns molecular dynamics simulations for each ligand through three independent simulations. According to MM/GBSA calculations, the binding affinity of the modified ligands targeting TEAD4 was similar to the original ligands. This suggested a new class of inhibitors targeting the TEAD4 central pocket in addition to the experimentally identified ligands.

The distribution of the complex simulations in the reduced dimensional space constructed using t-ICA method revealed that the TEAD4-YAP1 complex explores different regions in conformational space when bound with different ligands. This implied that the interactions between ligands and TEAD4 could mediate the distribution of the TEAD4-YAP1 complex in its conformational space. This regulatory role of protein conformation distribution is an important factor for further development of more effective inhibitors. Furthermore, the MSM based on the simulations of all systems was built mainly to reveal and compare the impact of ligand binding on the properties of the TEAD4-YAP1 complex. The consistent behavior of the simulation systems in the unified MSM suggested that the binding with various ligands leads to different conformational states but poses little impact on the kinetics of the TEAD4-YAP1 complex.

All 12 ligands under consideration displaying a stabilization effect on the TEAD4-YAP1 complex exhibited more favorable binding affinities between TEAD4 and YAP1 than the binding affinity of the TEAD4-YAP1 complex without any ligand. Four chemically modified ligands displayed an enhanced stabilization effect on the TEAD4-YAP1 complex compared to their corresponding original ligands. The inspection of interactions between TEAD4 and those ligands with more favorable binding affinities with TEAD4 and more favorable binding affinities of TEAD4-YAP1 complex revealed several key residues, including Phe229, Thr332, Ile374, and Ile395, which should be considered carefully for further development of the inhibitors against the TEAD4-YAP1 complex.

## 4. Materials and Methods

### 4.1. Molecular Docking of Original and Modified Molecules

Chemical structural modification of the six experimentally identified ligands from the screening of the DNA-encoding library was performed by replacing the secondary methyl amide with chloromethyl ketone, inspired by the chemical structure of TED-347 [6]. A conformational search was performed on the 12 ligands targeting the hydrophobic palmitate-binding pocket of TEAD4 protein using the knowledge-based method OMEGA2 [32]. A conformational search was carried out to find the most stable conformation of the 12 ligands by taking into consideration the ligand flexibility.

The crystal structure of the TEAD4-YAP1 complex (PDB ID: 5OAQ) was retrieved from the Protein Data Bank (PDB) [33]. The hydrophobic palmitate-binding pocket of TEAD4 protein was prepared for the molecular docking of the experimentally identified ligands and the modified ligands using the MakeReceptor package, which is part of the OpenEye Scientific software, Santa Fe, NM, USA [34]. MakeReceptor generates a negative image to represent the shape of the receptor’s active site. This negative image represents the space that the ligand can occupy without clashing in the target mask and can be used for docking purpose [34]. Molecular docking of the 12 ligands was performed against the negative image using the FRED package [27,28,29]. FRED arranges all the generated docked poses from the systematic search using the Chemgauss4 scoring function based on the shape complementarity of the docked ligands with the active site.

### 4.2. Molecular Dynamics Simulations

The top poses for each of the 12 ligands obtained from the molecular docking using the FRED package [27,28,29] were subjected to molecular dynamics simulations. These 12 ligands were parameterized using one auxiliary tool available in the AMBER molecular dynamics software package, version 22 (University of California, San Francisco, CA, USA) [35,36], AnteChamber [37,38]. General AMBER Forcefield 2 (GAFF2) parameters were generated for these ligands for simulations reported in this study.

Molecular dynamics simulations were carried out for the 12 protein–ligand complexes to evaluate their stability and to explore the flexibility of both protein and ligands. The AMBER package was employed for the molecular dynamics simulations using AMBER ff14SB force field for the protein [39,40,41]. The protein–ligand complexes were solvated using the TIP3P water model in cubic water boxes with the minimum 10.0 Å between any atom of the protein–ligand complex and the edge of the simulation box. Counter Na^+^ and Cl^−^ ions were added to neutralize the solvated protein–ligand complexes, followed by the minimization of the constructed systems using the steepest descent algorithm. For each protein–ligand complex, three independent replicas were generated and subjected to 50 ps equilibration simulation at 300 K using an isothermal-isobaric ensemble (NPT). A 50 ns simulation using canonical ensemble (NVT) was carried out for each independent replica following the NPT equilibration simulation, resulting in a total of 150 ns simulations for each protein–ligand complex. Similarly, a total of 150 ns simulations using NVT were generated for the TEAD4-YAP1 complex without ligand following the same protocol. The simulation trajectories were saved for every 100 ps for all production runs. The covalent bonds including a hydrogen atom were constrained during simulation using the SHAKE algorithm [42]. The electrostatic potentials were calculated using the particle-mesh Ewald method [42]. A cutoff value of 8.0 Å was applied for van der Waals interactions using the Lennard–Jones potential. It is noteworthy that a recent study demonstrated that Reaction Field can serve as a viable alternative to PME for long-range electrostatics in relative free energy calculations, exhibiting comparable efficiency [43].

The stability of the binding complexes with the 12 ligands bound to the TEAD4 hydrophobic pocket was assessed using the root mean square deviation (RMSD) with respect to the first frame of the simulation calculated using Equation (1) implemented in AmberTools22 [44]

Root-mean-square fluctuation (RMSF) was also calculated using AmberTools22 [44] to account for the fluctuations of the atoms over the time course of the simulation with respect to the apo crystal structure of the TEAD4-YAP1 complex. The RMSF was calculated through Equation (2).

### 4.3. MM/GBSA Binding Energy Calculations

Molecular mechanics with the generalized Born and surface area solvation (MM/GBSA) method [45] was applied to calculate the binding free energy of the 12 ligands to the TEAD4 palmitate-binding pocket using AmberTools22 [44]. In the MM/GBSA method, binding free energy between protein and ligand was estimated using Equation (3) based on hypothetical thermodynamics cycles using the protein, ligand, and their complex in both solvated and vacuum states. This was estimated by including the protein–ligand nonbonded interaction (the MM part calculated by including the van der Waals and electrostatic interactions), the solvated electrostatic contribution (GB part), and the solvated hydrophobic contribution (SA part).

The MM/GBSA may not possess the same robustness as other molecular dynamics (MD)-based methods such as free energy perturbation, which is commonly used for calculating ligand binding free energies [46]. However, it remains well suited for comparing the binding affinities of the experimentally identified ligands and modified ligands in this work. Several studies have found MM/GBSA to be a suitable approach for subsequent free energy calculations [47].

The MM/GBSA method was also used to calculate the difference of the TEAD4-YAP1 binding free energy between the bound state with ligand and unbound state without ligand (ΔΔG) to evaluate the effect of the ligands’ binding on the strength of the protein–protein interaction between TEAD4 and YAP1.

### 4.4. Analysis of Conformational Space

#### 4.4.1. Time-Independent Component Analysis

Pairwise backbone Cα distances from the 13 generated molecular dynamics simulations were used as the input geometric features for the dimensionality reduction technique time-independent component analysis (t-ICA) implemented in MSMBuilder [48]. In other words, the Cartesian coordinates of the protein structures generated from the simulations were transformed into orientation-invariable generalized coordinates of the system. The t-ICA method was developed to reveal the slowest degrees of freedom by diagonalizing the time-lagged covariance matrix and comparing the eigen values of eigen vectors related to protein motions [49]. Separate t-ICA analyses for each configuration of the original and modified molecules have been performed (Figures S5 and S6). 

#### 4.4.2. Markov State Model Analysis

Markov state model (MSM) was built to divide the conformational space of the trajectories into distinct macrostates using MSMBuilder [48]. In the MSM, a transition probability matrix was provided based on the number of transitions among macrostates observed in the simulation using Equation (4). Perron cluster–cluster analysis (PCCA) was used to generate the distinct macrostates. This dynamical clustering to obtain kinetically meaningful macrostates was achieved by the eigenvalue decomposition of the transition probability matrix (Equation (5)). The suitable lag time and the corresponding number of macrostates were selected based on the convergence of the implied relaxation timescale. A total of eight macrostates were selected for the simulations of the TEAD4-YAP1 complex with the ligands because of the apparent gap below the top seven lines of the implied time scale plot (Figure 6a). The Generalized Matrix Rayleigh Quotient (GMRQ) was employed to assess the quality of the constructed Markov state model (MSM). A higher GMRQ score indicated a better MSM model, as it signified an enhanced ability to identify and capture the slowest dynamical processes within the simulations [49].

## 5. Conclusions

Developing allosteric inhibitors against the TEAD4 central hydrophobic pocket is an effective approach to regulating TEAD4-YAP1 interaction. Screening of the DNA-encoded library resulted in six potent ligands against the TEAD4 central pocket. Inspired by a previous report, the secondary methyl amide in the original ligands was replaced by a chloromethyl ketone moiety. TEAD4-YAP1 complexes bound with both original and chemically modified ligands were subjected to molecular dynamics simulations and subsequent analyses. The computational results indicated that the binding with all 12 ligands under study could stabilize the TEAD4-YAP1 complex. The chemical modification of the original inhibitors led to the enhanced stabilization effect of the TEAD4-YAP1 complex for four ligands. Analysis of local interactions between ligands and TEAD4 revealed residues that interacted with the ligands and could play a key role in regulating TEAD4-YAP1 complex dynamics through binding with ligands. Overall, this study provides dynamical and structural insight into the binding and regulation effect of different inhibitors on TEAD4-YAP1 interaction.

## Figures and Tables

**Table 1:** Chemgauss4 docking scores of the experimentally identified inhibitors and the modified inhibitors obtained from the FRED package [27,28,29].

Ligands	R1 ^a^	Chemgauss4 Docking Score (kcal/mol)	
		Original	Modified
1	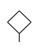	−9.04	−8.24
2	-SF3	−8.16	−8.99
3	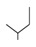	−9.16	−9.18
4	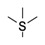	−7.82	−8.16
5	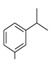	−9.67	−10.64
6	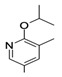	−7.90	−8.84

^a^ Please refer to Figure 2 for ligands including the R1 substituent group.

**Table 2:** The binding free energies of the original experimentally identified and the computationally modified ligands calculated by the MM/GBSA method.

Ligands	R1 ^a^	ΔG (kcal/mol)	
		Original	Modified
1	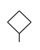	−62.22 ± 2.94	−56.90 ± 3.65
2	-SF3	−56.33 ± 3.50	−49.87 ± 3.17
3	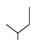	−65.34 ± 3.13	−62.73 ± 3.84
4	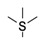	−57.87 ± 4.97	−58.18 ± 2.46
5	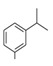	−63.99 ± 3.15	−60.20 ± 2.48
6	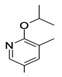	−60.88 ± 3.40	−56.07 ± 3.62

^a^ Please refer to Figure 2 for ligands including R1 substituent group.

## Data Availability

The crystal structure of the TEAD4-YAP1 complex (PDB ID: 5OAQ) can be retrieved free of charge from the Protein Data Bank (https://www.rcsb.org/, accessed on 18 August 2022). MakeReceptor module, Omega, and FRED can be obtained from OpenEye Scientific using (https://www.eyesopen.com, accessed on 18 August 2022).

## Supplementary Materials

The following supporting information can be downloaded at: https://www.mdpi.com/article/10.3390/ijms24109009/s1.
