# Supplementary material for: Novel Allosteric Effectors Targeting Human Transcription Factor TEAD
Source: Int J Mol Sci. 2023 May 19;24(10):9009. doi: 10.3390/ijms24109009 (PMC10219411; doi:10.3390/ijms24109009)
Supplement: Supplementary file 1 [file ijms-24-09009-s001.zip › ijms-2350474-supplementary.pdf]

Supporting Information

NOVEL ALLOSTERIC EFFECTORS TARGETING HUMAN TRANSCRIPTION TEAD

Mayar Tarek Ibrahim[1], Gennady M. Verkhivker[*2,3], Jyoti Misra[4], and Peng Tao[*1]

[1] Department of Chemistry, Center for Research Computing, Center for Drug Discovery, Design, and Delivery (CD4), Southern Methodist University, Dallas, TX 75205, USA; mayarm@smu.edu (M.T.I.); ptao@smu.edu (P.T.)
[2] Graduate Program in Computational and Data Sciences, Schmid College of Science and Technology, Chapman University, Orange, CA 92866, USA
[3] Department of Biomedical and Pharmaceutical Sciences, Chapman University School of Pharmacy, Irvine, CA 92618, USA
[4] Department of Biological Sciences, University of Texas at Dallas, Richardson, TX 75080, USA
* Correspondence: verkhivk@chapman.edu

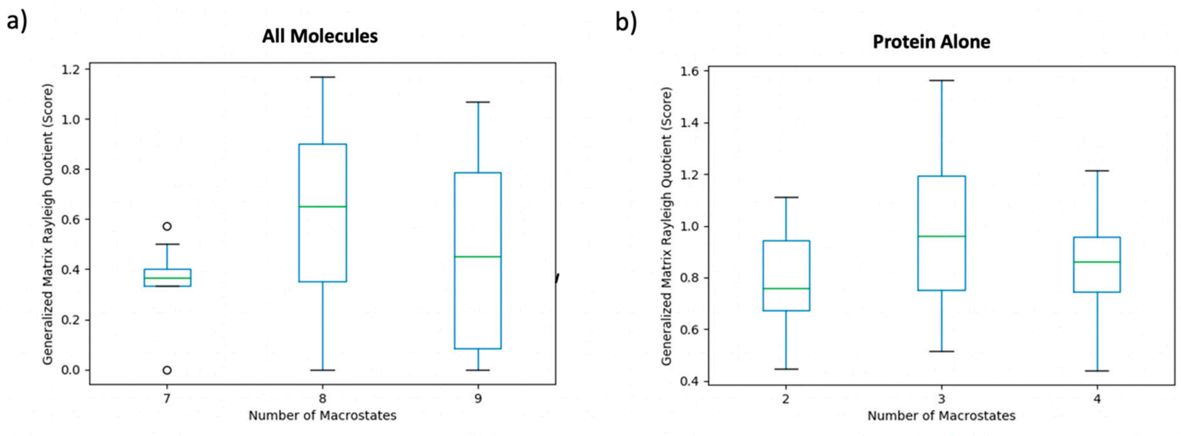

Figure S1: GMRQ scores against the different number of macrostates for the apo structure and the holo structure of the protein. a) 8 macrostates were associated with the highest GMRQ score for the holo structure model. Outliers in macrostate 7 plot are represented by circles; b) 3 macrostates were associated with the highest GMRQ score for the apo structure model.

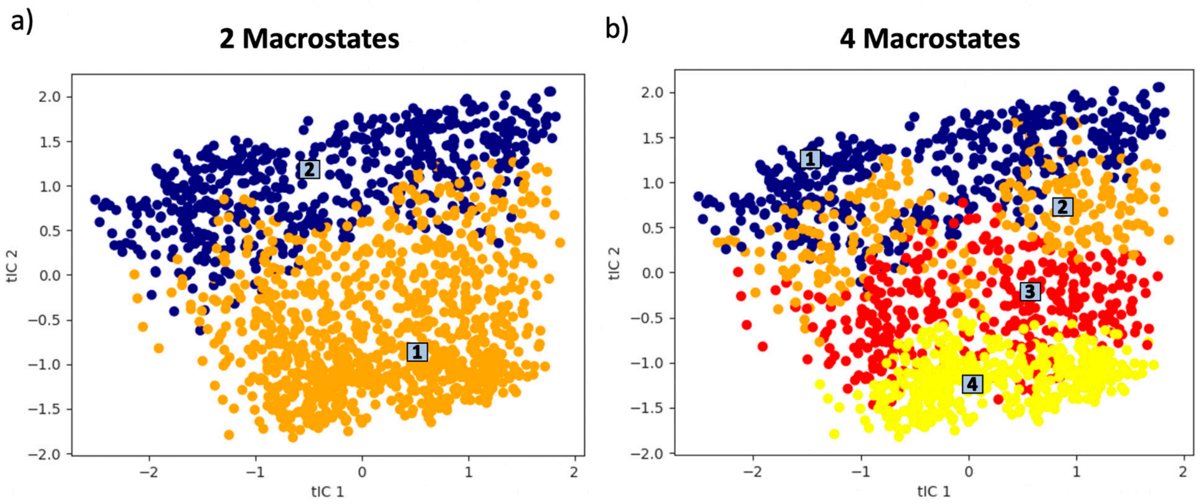

Figure S2: Different number of macrostates for the apo structure of the protein were explored to find the best separation in the corresponding conformational space from MSMBuilder [1]. a) The conformational space of the 2 macrostates generated from the trajectories of the apo structure of TEAD4/YAP1 complex; b) The conformational space of the 4 macrostates generated from the trajectories of the apo structure. The identified macrostates are numbered and colored differently for clarity.

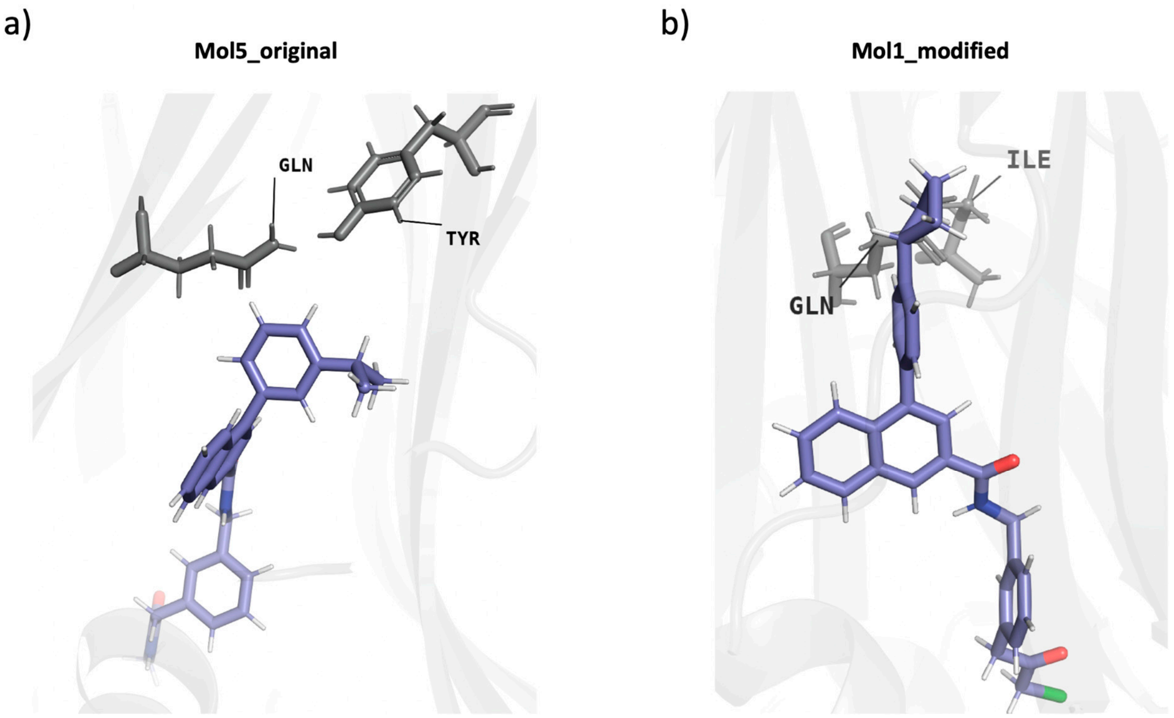

Figure S3: Comparison of the interactions associated with Gln397 between mol5 and other ligands. (a) Hydrogen bond between Gln397 and Tyr320 in mol5 complex represented by mol5_original. (b) Hydrogen bond between Gln397 and Ile411 in other systems represented by mol1_modified complex.

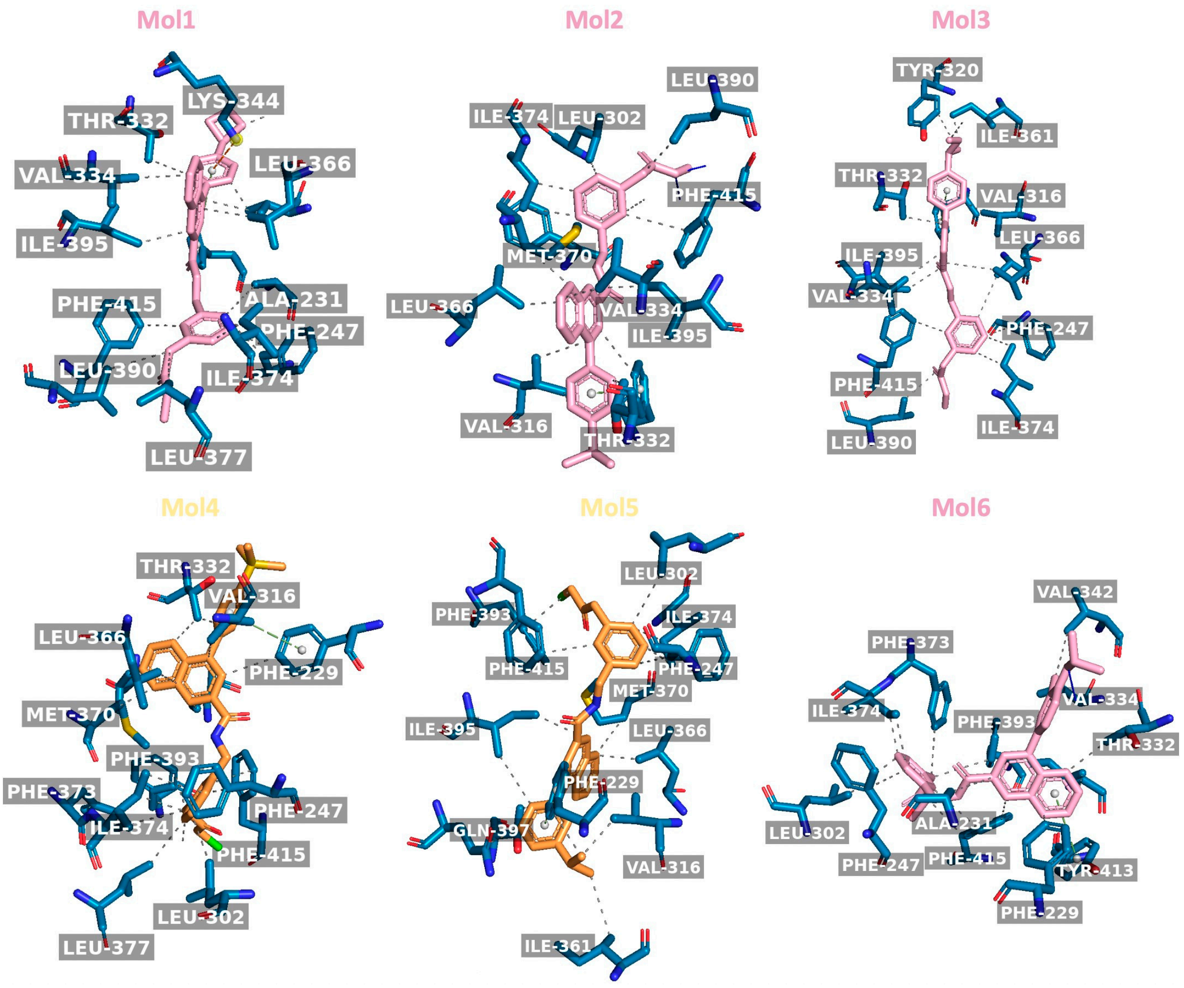

Figure S4: The binding mode of the molecules associated with a lower change in the DDG values, generated using PLIP package [2]. The original molecules colored in pink and the modified molecules colored in yellow.

Original Molecules

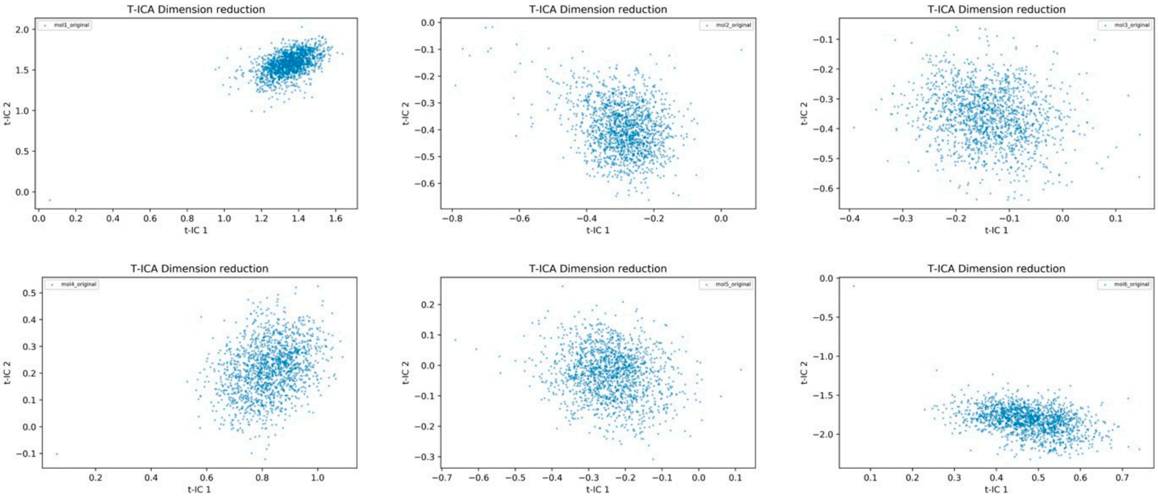

Figure S5: Separate t-ICA analysis for each configuration of the experimentally identified molecules.

Modified Molecules

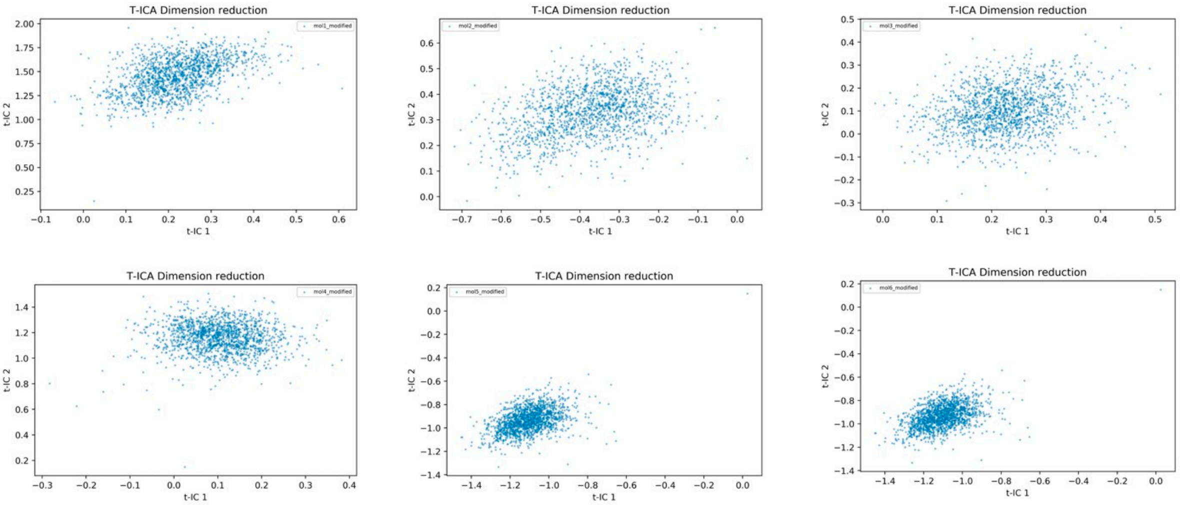

Figure S6: Separate t-ICA analysis for each configuration of the modified molecules.

Table S1: The average binding affinity between TEAD4 and YAP1 calculated from three independent replicas. The free energy was calculated in the case of both the holo structures, with original and computationally modified ligands bound, and the apo structures, without ligand bound.

Ligands	ΔG (kcal/mol)		ΔΔG (kcal/mol)	
	Original	Modified	Original	Modified
1	-118.39	-121.68	-18.43	-21.72
2	-100.37	-114.68	-0.41	-14.72
3	-115.08	-121.93	-15.12	-21.98
4	-124.39	-106.49	-24.44	-6.54
5	-119.90	-112.26	-19.94	-12.31
6	-107.67	-116.93	-7.72	-16.98
No ligand	-99.9549			

References

[1] M. P. Harrigan *et al.*, "MSMBuilder: statistical models for biomolecular dynamics," *Biophys J*, vol. 112, no. 1, pp. 10–15, 2017.

[2] S. Salentin, S. Schreiber, V. J. Haupt, M. F. Adasme, and M. Schroeder, "PLIP: fully automated protein–ligand interaction profiler," *Nucleic Acids Res*, vol. 43, no. W1, pp. W443–W447, 2015.
